# Toll-like receptor 7 governs interferon and inflammatory responses to rhinovirus and is suppressed by IL-5-induced lung eosinophilia

**DOI:** 10.1136/thoraxjnl-2014-205465

**Published:** 2015-06-24

**Authors:** Luke Hatchwell, Adam Collison, Jason Girkin, Kristy Parsons, Junyao Li, Jie Zhang, Simon Phipps, Darryl Knight, Nathan W Bartlett, Sebastian L Johnston, Paul S Foster, Peter A B Wark, Joerg Mattes

**Affiliations:** 1 Department of Experimental & Translational Respiratory Medicine, Hunter Medical Research Institute, University of Newcastle, Newcastle, New South Wales, Australia; 2 Priority Research Centre for Asthma and Respiratory Diseases, Hunter Medical Research Institute, University of Newcastle, Newcastle, New South Wales, Australia; 3 Department of Respiratory and Sleep Medicine, John Hunter Hospital, Newcastle, New South Wales, Australia; 4 Norman Bethune Medical Science Centre, Jilin University, Jilin, Changchun, China; 5 The School of Biomedical Sciences, University of Queensland, Queensland, Queensland, Australia; 6 Airway Disease Infection Section, National Heart and Lung Institute, Medical Research Council & Asthma UK Centre in Allergic Mechanisms of Asthma, Imperial College London, Norfolk Place, London, UK; 7 Department of Paediatric Respiratory and Sleep Medicine, Newcastle Children's Hospital, Newcastle, New South Wales, Australia

**Keywords:** Asthma, Respiratory Infection, Pulmonary eosinophilia, Innate Immunity

## Abstract

**Background:**

Asthma exacerbations represent a significant disease burden and are commonly caused by rhinovirus (RV), which is sensed by Toll-like receptors (TLR) such as TLR7. Some asthmatics have impaired interferon (IFN) responses to RV, but the underlying mechanisms of this clinically relevant observation are poorly understood.

**Objectives:**

To investigate the importance of intact TLR7 signalling in vivo during RV exacerbation using mouse models of house dust mite (HDM)-induced allergic airways disease exacerbated by a superimposed RV infection.

**Methods:**

Wild-type and TLR7-deficient (*Tlr7^−/−^*) BALB/c mice were intranasally sensitised and challenged with HDM prior to infection with RV1B. In some experiments, mice were administered recombinant IFN or adoptively transferred with plasmacytoid dendritic cells (pDC).

**Results:**

Allergic *Tlr7^−/−^* mice displayed impaired IFN release upon RV1B infection, increased virus replication and exaggerated eosinophilic inflammation and airways hyper reactivity. Treatment with exogenous IFN or adoptive transfer of TLR7-competent pDCs blocked these exaggerated inflammatory responses and boosted IFNγ release in the absence of host TLR7 signalling. TLR7 expression in the lungs was suppressed by allergic inflammation and by interleukin (IL)-5-induced eosinophilia in the absence of allergy. Subjects with moderate-to-severe asthma and eosinophilic but not neutrophilic airways inflammation, despite inhaled steroids, showed reduced TLR7 and IFNλ2/3 expression in endobronchial biopsies. Furthermore, TLR7 expression inversely correlated with percentage of sputum eosinophils.

**Conclusions:**

This implicates IL-5-induced airways eosinophilia as a negative regulator of TLR7 expression and antiviral responses, which provides a molecular mechanism underpinning the effect of eosinophil-targeting treatments for the prevention of asthma exacerbations.

**Key messages**

**What is the key question?**

- What underlies impaired interferon responses to rhinovirus in asthmatics.

**What is the bottom line?**

- Suppression of Toll-like receptor (TLR)7 by interleukin-5-induced lung eosinophilia impairs interferon responses to rhinovirus, leading to exaggerated inflammatory responses and exacerbation.

**Why read on?**

- This provides a mechanism whereby therapies modulating TLR7 or targeting eosinophils may ameliorate virus-induced asthma exacerbations.

## Introduction

Asthma is a complex and heterogenic inflammatory disease of the airways with increasing global prevalence. The most common trigger for asthma symptoms is immune cell activation against innocuous antigens (allergens) and respiratory viral infections.^1^ Upon antigen exposure, cytokines such as thymic stromal lymphopoietin (TSLP), granulocyte-macrophage colony-stimulating factor, interleukin (IL)-25, IL-33 and tumour necrosis factor-related apoptosis-inducing ligand are released by the airway epithelium, resulting in the activation of innate immune cells that promote T helper 2 (T_H_2) cell differentiation, leading to the release of T_H_2 cytokines such as IL-4, IL-5 and IL-13.^1^ ^2^ IL-13 is a potent inducer of airway hyper reactivity (AHR) and mucus production in a signal transducer and activator of transcription-6 (STAT6)-dependent manner.^3^ IL-5 regulates maturation of eosinophils in the bone marrow and in concert with chemokines such as eotaxins the recruitment of these cells into the airways.^4^ Thus, T_H_2 cell activation underpins many clinical phenotypes including allergic and eosinophilic asthma.^5^ The proportion of asthmatics with high eosinophil numbers in their airways represent the majority of patients, and some of those unresponsive to corticosteroids have severe therapy-refractory asthma with a disproportionally large burden of disease. Importantly, they are also prone to asthma exacerbations, which can be partially alleviated by therapeutics that block IL-4, IL-5 or IL-13 and reduce eosinophilic inflammation in the lungs.^5^ However, the molecular mechanisms that link T_H_2-induced eosinophilia with susceptibility to exacerbation are yet to be defined.

Viral respiratory infections are detected in up to 85% of asthma exacerbations and two-thirds of those are caused by rhinoviruses (RV).^6^ Notably a group of asthmatics have impaired release of innate interferons (IFNα, IFNβ and IFNλ) upon experimental RV infection and IFNλ inversely correlated with induced sputum eosinophils on day 3 of acute infection.^7^ ^8^ IFNλ 2/3 inversely correlated with induced sputum eosinophils on day 3 of the acute infection (r=−0.53, p=0.05), when subjects were experimentally infected with RV16 in vivo.^8^ RV is sensed by a limited number of pattern recognition receptors, including Toll-like receptors (TLRs) and retinoid acid-induced gene 1 like receptors. TLR3 and TLR7 are endosomally localised and recognise viral nucleic acids, critically regulating antiviral IFN production. Recent studies have shown that impaired TLR3 function does not affect RV replication in vivo and TLR3 expression is not reduced in asthmatics.^9^ ^10^ The in vivo role of functional TLR7 signalling in mounting antiviral responses to RV has yet to be determined. TLR7 is widely expressed in innate immune cells such as dendritic cells (DCs) and macrophages, as well as in structural lung cells, including airway epithelia.^11^ Treatment of mice with TLR7 agonists leads to a long-lasting protection from the development of allergic airways disease (AAD) and TLR7 activation also reduced airway smooth muscle contractility in mice.^12^ We and others have found reduced IFNα and IFNλ release upon TLR7 stimulation of peripheral blood mononuclear cells from asthmatics and a trend towards lower TLR7 expression was observed in bronchoalveolar lavage fluid (BALF) cells from asthmatics.^9^ However, the role of TLR7 in RV-induced exacerbation of AAD has yet to be fully elucidated.

## Methods

### Patient biopsies

Endobronchial biopsies were obtained by bronchoscopy with samples taken from third-generation bronchi. Biopsies were stored in RNALater (Ambion) at −20°C until needed. RNA was extracted following the miRNeasy Mini Handbook (Qiagen)—purification of total RNA from tissue via homogenisation (Qiashredder). RNA was quantified by spectrophotometry (NanoDrop) and 200 ng of extracted RNA reverse transcribed using High Capacity cDNA Reverse Transcription Kit (Applied Biosystems; ABI). The inflammatory phenotype was determined by induced sputum count, and patients with a RAST ImmunoCAP Specific IgE-positive blood test were designated as atopic.

### Animals

Wild-type (WT), *Tlr7*^−/−^, *Tlr4*^−/−^, *MyD88*^−/−^, *Stat6^−/−^* and *IL-5* transgenic mice, all on a BALB/c background (6–14 weeks of age), were obtained from Australian BioResources (Moss Vale, Australia) and housed in approved containment facilities within the HMRI Building, University of Newcastle (Newcastle, Australia). Mice had ad libitum access to food and water under a 12 h light and dark cycle. All experiments were approved by the Animal Care and Ethics Committee of the University of Newcastle.

### Induction of AAD and RV-induced exacerbation

AAD and RV-induced exacerbation were performed as previously described.^13^ Briefly, lyophilised crude house dust mite (HDM) (*Dermatophagoides pteronyssinus*) extract (Greer Laboratories) was resuspended in sterile saline (SAL) and intranasally delivered to mice under light isoflurane anaesthesia. Sensitisation on days 0, 1 and 2 (HDM 50 µg/50 µL) was followed by daily challenge (HDM 5 µg/50 µL) on days 14, 15, 16 and 17 to induce AAD. On day 18, mice were euthanased via pentobarbital sodium overdose (Virbac) and samples collected. In other experiments, mice were intranasally infected with live minor group RV (RV1B), 50 µL containing 5×10^6^ virions median tissue culture infective dose (TCID_50_) or UV-inactivated RV1B 24 h after last HDM exposure to exacerbate pre-existing AAD. Samples were collected 24 h after RV1B infection.

### Administration of recombinant cytokines and LPS

Naive mice were intranasally administered recombinant mouse IL-13 (15 µg/35 µL; Biolegend), IL-5 (15 µg/35 µL; Biolegend) or a low or high dose (0.12 µg/50 µL and 10 µg/50 µL ,respectively) of lipopolysaccharide (LPS) (*Escherichia coli*, 0111:B4; Sigma-Aldrich), with samples collected 24 h later. HDM-sensitised and challenged *Tlr7*^−/−^ mice, which also received live RV1B, were intranasally administered either recombinant mouse IFNα2 (10 000 IU/50 µL; eBioscience), IFNβ1 (10 000 IU/50 µL; Biolegend), IFNλ2 (1 µg/50 µL; R&D Systems)^14^ or a vehicle control (phosphate buffered saline) on day 18 (2 h following RV1B infection). Mice were sacrificed 24 h postinfection and samples collected.

### AHR measurement

AHR was measured as previously described.^15^ Briefly, AHR was invasively assessed in separate groups of anaesthetised mice by measurement of total lung resistance and dynamic compliance (Buxco). Mice were mechanically ventilated, and AHR to nebulised methacholine (increased lung resistance) was expressed as a percentage change from control (baseline).

### Analysis of lung inflammation

BALF was collected and analysed as previously described.^16^ Enumeration of peribronchial/perivascular eosinophils and PAS-positive cells was performed as previously described.^17^

### Flow cytometry

Single lung cell suspensions were prepared and stained as previously described.^18^ Antibodies used were FITC-anti-TCRβ chain (BD, cat. no. 553171, clone H57-597), PE-anti-CD4 (BD, cat. no. 553652, clone H129.19), PerCP-anti-CD8a (BD, cat. no. 561092, clone 53-6.7), PerCP-Cy5.5-anti-CD11b (BD, cat. no. 561092, clone M1/70), FITC-anti-CD11c (BD, cat. no. 553801, clone HL3), PE-anti-MHCII (eBioscience, cat. no. 12-5321, clone M5/114.15.2), all at 1:15 dilution. Positive cells were identified using a FACSCanto (BD) by the following criteria: mDCs—CD11b^+^ CD11c^+^ MHCII^+^; T cells—TCRβ chain^+^ with CD4^+^ or CD8a^+^. Data analysed with BD FACsdiva.

### Quantitative RT-PCR

Trachea and lungs were extracted from euthanased mice and forceps used to separate the airways from the parenchyma by blunt dissection.^19^ Total mRNA was extracted using TRIzol (Ambion; Carlsbad, USA). cDNA was generated via reverse transcription using BioScript (Bioline; Alexandria, Australia). Quantitative PCRs (qPCR) were performed on cDNA generated from mouse airway tissue and human endobronchial biopsies with SYBR Green (Invitrogen; Mulgrave, Australia) using primers detailed in online supplementary table S1. CTs of the genes of interest were referenced HPRT or GAPDH for mouse and human tissue, respectively. All steps were performed according to manufacturer's instructions.

### Generation of BM-derived pDCs and adoptive transfer

WT and TLR7-deficient bone marrow-derived plasmacytoid DCs (pDCs) were generated as previously described.^20^ pDCs were resuspended at 1×10^7^ cells/mL and 0.5×10^6^ pDC were administered intranasally to allergic *Tlr7^−/−^* mice, 2 h prior to RV1B infection.

### Quantification of lung cytokines and IFNs

A single lung lobe from each mouse was excised and snap frozen before being homogenised in buffers recommended in the manufacturer's instructions. Levels of IL-5, IL-13, IFNγ (BD Biosciences; PharMingen), IFNα, IFNβ (Verikine; PBL Assay Science), CCL7/MCP3 and IFNλ2/3 (R&D Systems) were determined in clarified lung lysates by ELISA. CCL2/MCP1, CCL3/MIP1α, CCL4/MIP1β, CXCL9/MIG and CXCL10/IP10 were measured by employing a Multiplex Immunoassay (Millipore). All concentrations were normalised to lung weight.

### pDC culture and TCID_50_

Primary mouse pDCs were isolated from pooled WT or TLR7-deficient spleens via mechanical dissociation and isolation with PDCA-1 magnetic beads on an AutoMACS platform (Miltenyi Biotec) according to manufacturer's instructions. Following two isolation runs, positively selected cells were seeded into 96-well plates at 1×10^6^ cells/well and cultured in the absence or presence of RV1B (multiplicity of infection =5).^20^ Supernatant was harvested 48 h postinfection and IFN production assessed with ELISA. Number of live RV1B virions was determined by standard TCID_50_ serial dilution on Hela-H1 cells, calculated via the Spearman–Karber method.

### Statistical analysis

The significance of differences between groups was analysed using Student's t test, Mann–Whitney test, analysis of variance or Kruskal–Wallis with Dunn's test for multiple comparisons as appropriate using Graphpad Prism 6. A value of p<0.05 is reported as significant. Data are expressed as mean±SEM.

## Results

**Figure 1:**
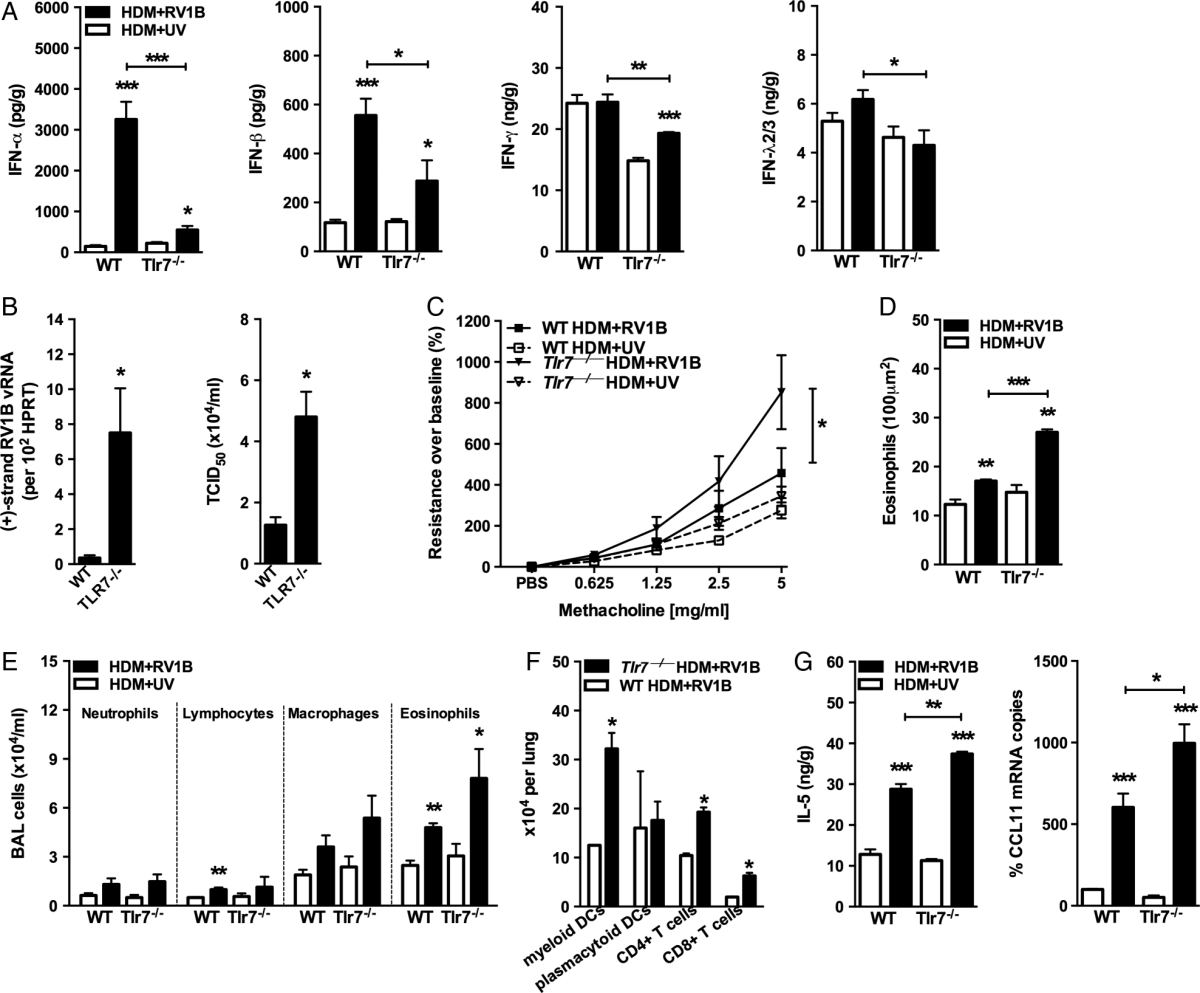
Toll-like receptor (TLR)7-deficient mice experience exaggerated rhinovirus (RV)-induced exacerbation. Allergic wildtype (WT) and TLR7-deficient mice were inoculated with live or UV-inactivated RV1B, samples collected 24 h post infection. Levels of interferons (A) and interleukin (IL)-5 (G) in clarified lung homogenates as assessed by ELISA. (B) Lung viral titre in infected mice was quantified via quantitative RT-PCR (qRT-PCR) and 50% tissue culture infectious dose (TCID_50_). (C) Total lung resistance presented as percentage change in response to methacholine (n=4–6 mice per group). Peribronchial/perivascular eosinophils and cellular infiltrates in bronchoalveolar lavage (BAL) fluid enumerated by light microscopy (D and E). (F) Numbers of lung mDCs and CD4+/CD8+ T cells quantified by flow cytometry. (G) CCL11 expression in lower airway tissue enumerated by qRT-PCR and expressed as % increase over WT house dust mite (HDM)+UV group. Results are mean±SEM (n=3–6 mice per group) and are representative of two independent experiments. *p<0.05, **p<0.01, ***p<0.001 as compared to strain-matched HDM+UV group or otherwise indicated determined by students t test except for (C) where analysis of variance was used to compare RI curves.

To investigate TLR7 as a regulator of AAD and antiviral responses in vivo, we repeatedly challenged WT and TLR7-deficient (*Tlr7***^−/−^**) mice with HDM, then superimposed a RV infection to exacerbate their established AAD as described previously.^15^ Exposure of allergic *Tlr7***^−/−^** mice to RV, compared with WT controls, resulted in impaired production of type I (α, β), II (γ) and III (λ) IFNs with higher RV replication in the lower airways (figure 1A,B). This was despite a lack of an increase in type II (γ) and III (λ) IFNs to RV infection in allergic WT mice. Deficient IFN responses coincided with exaggerated AHR (figure 1C), eosinophilic airways inflammation (figure 1D,E), accumulation of CD4+, CD8+ and myeloid (m) DCs but not pDCs in the lungs (figure 1F), and production IL-5 and CCL11 (eotaxin-1) (figure 1G). We also observed increased levels of the T_H_2-priming cytokines IL-25 and TSLP in allergic *Tlr7***^−/−^** mice infected with RV (see online supplementary figure S1). CCL2, CCL3, CCL4 and CCL7 but not CXCL9 and CXCL10 were also increased in the absence of TLR7 signalling (see online supplementary figure S2). There was no change in IL-13 levels or numbers of mucus-producing cells (see online supplementary figure S3a), suggesting a novel and critical role of TLR7 signalling in RV-induced asthma exacerbation.

**Figure 2:**
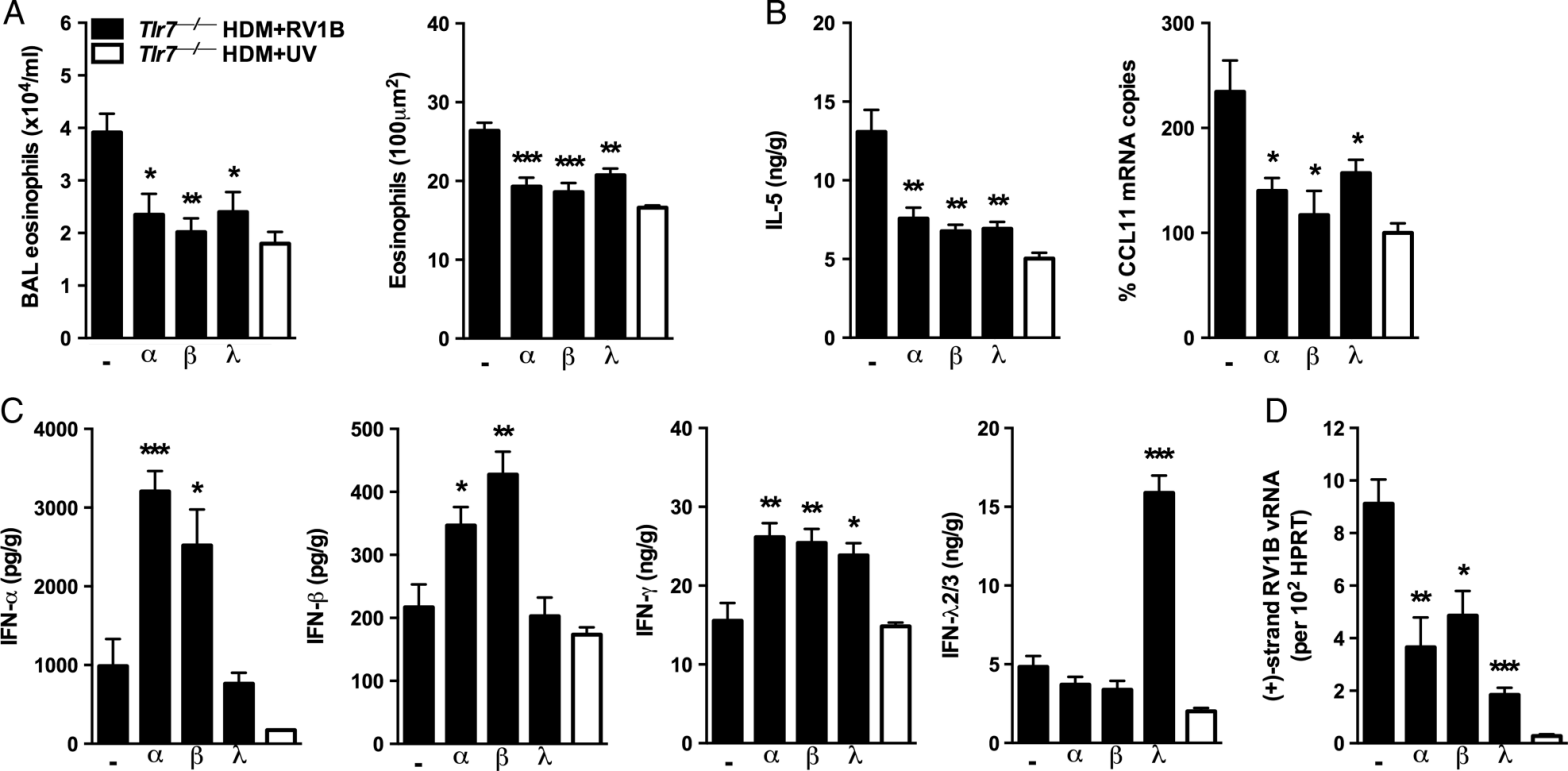
Exogenous interferon can protect Toll-like receptor (TLR)7-deficient mice from rhinovirus (RV)-induced exacerbation. Exacerbated TLR7-deficient mice received recombinant interferon (IFN)α2 (α), IFNβ (β), IFNλ2 (λ) or a vehicle control (−) 2 h post-RV1B infection on day 18. Samples were collected 24 h post infection. (A) Eosinophils present in bronchoalveolar lavage (BAL) fluid and per 100 µm^2^ of lung tissue. Levels of interleukin (IL)-5 (B) and interferons (C) in clarified lung homogenates as assessed by ELISA. CCL11 expression (B) and viral RV1B RNA (D) in lower airway tissue was quantified by quantitative RT-PCR. Results are mean±SEM (n=3–7 mice per group) and are representative of two independent experiments. *p<0.05, **p<0.01, ***p<0.001 as compared to vehicle control group determined by Student's t test.

Allergic *Tlr7***^−/−^** mice received recombinant type I and III IFNs 2 h after infection with RV. Notably, one dose of IFNα2, β or λ2 suppressed RV-induced eosinophilic inflammation (figure 2A), as well as IL-5 and CCL11 production (figure 2B) but not IL-13 (see online supplementary figure S3b). All IFN treatments induced IFN-γ in the lung (figure 2C) and limited RV replication (figure 2D). There was no effect of IFN treatment on TSLP, IL-25 or IL-33 expression (data not shown). Thus IFN treatment promotes IFN-γ release and impairs IL-5 and CCL11 production and RV replication in the absence of TLR7.

**Figure 3:**
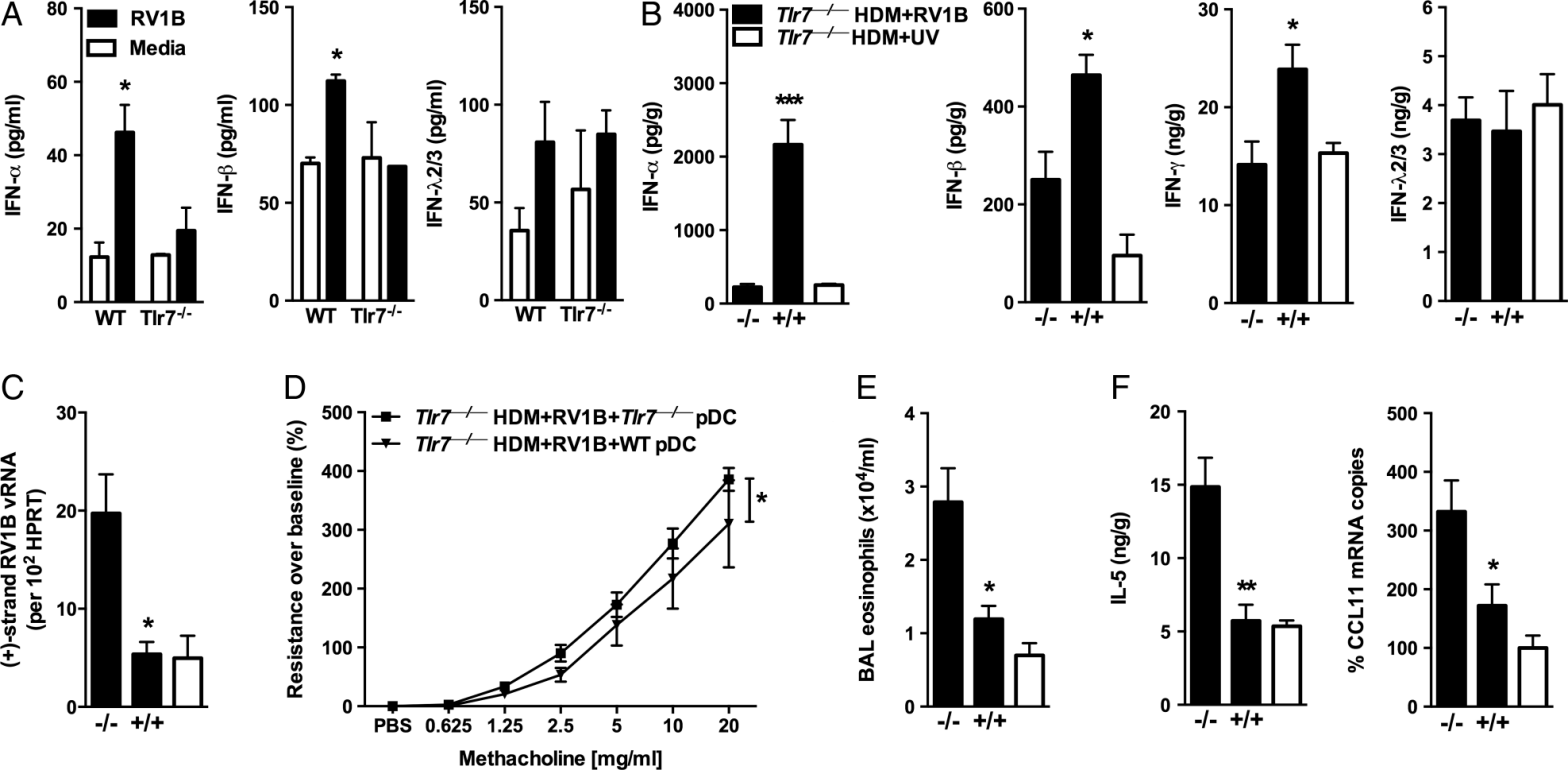
Adoptive transfer of Toll-like receptor (TLR)7-competent plasmacytoid dendritic cells (pDCs) to TLR7-deficient mice limits exacerbation of allergic airways disease (AAD). Purified Flt3-L-expanded pDCs from TLR7-deficient (−/−) and TLR7-competent (+/+) bone marrow were adoptively transferred to allergic *Tlr7*^−/−^ recipients. Mice were inoculated with RV1B 2 h later and endpoints measured 24 h post infection. (A) Spleen-isolated pDCs were infected in vitro with RV1B and interferon (IFN) release in cell supernatants assessed by ELISA. Levels of IFNs (B), as well as interleukin (IL)-5 (F) in clarified lung homogenates as assessed by ELISA. Positive-strand RV1B RNA (C) and CCL11 expression (F) from lower airway tissue quantified by quantitative RT-PCR. (D) Total lung resistance presented as percentage change in response to methacholine (n=7–8 mice per group). (E) Eosinophils present in bronchoalveolar lavage (BAL) fluid. Results are mean±SEM (n=3–5 mice per group). *p<0.05, **p<0.01, ***p<0.001 determined by Student's t test except for (D) where analysis of variance compared the RI curves. All p values as compared to exacerbated mice that received TLR7-deficient pDCs.

pDCs release large quantities of IFN during viral infection, and here we show that splenic pDCs exposed to RV in vitro display impaired release of IFN-α and IFN-β but not λ2/3 in the absence of TLR7 (figure 3A). Adoptive transfer of TLR7-expressing pDCs into allergic *Tlr7*^−/−^ mice re-established IFNα, IFNβ and IFNγ but not IFNλ release in the lungs upon RV infection and limited RV replication as compared to allergic *Tlr7*^−/−^ mice that received TLR7-deficient pDCs (figure 3B, C). Transfer of WT pDCs also limited RV-induced AHR (figure 3D), eosinophilic airways inflammation (figure 3E) and production of IL-5 and CCL11 (figure 3F) but not IL-13 release (see online supplementary figure S3c). Thus adoptive transfer of TLR7-expressing pDCs—like exogenous IFN treatment—promotes IFNγ release and impairs eosinophilic airways inflammation and RV replication in the absence of TLR7.

**Figure 4:**
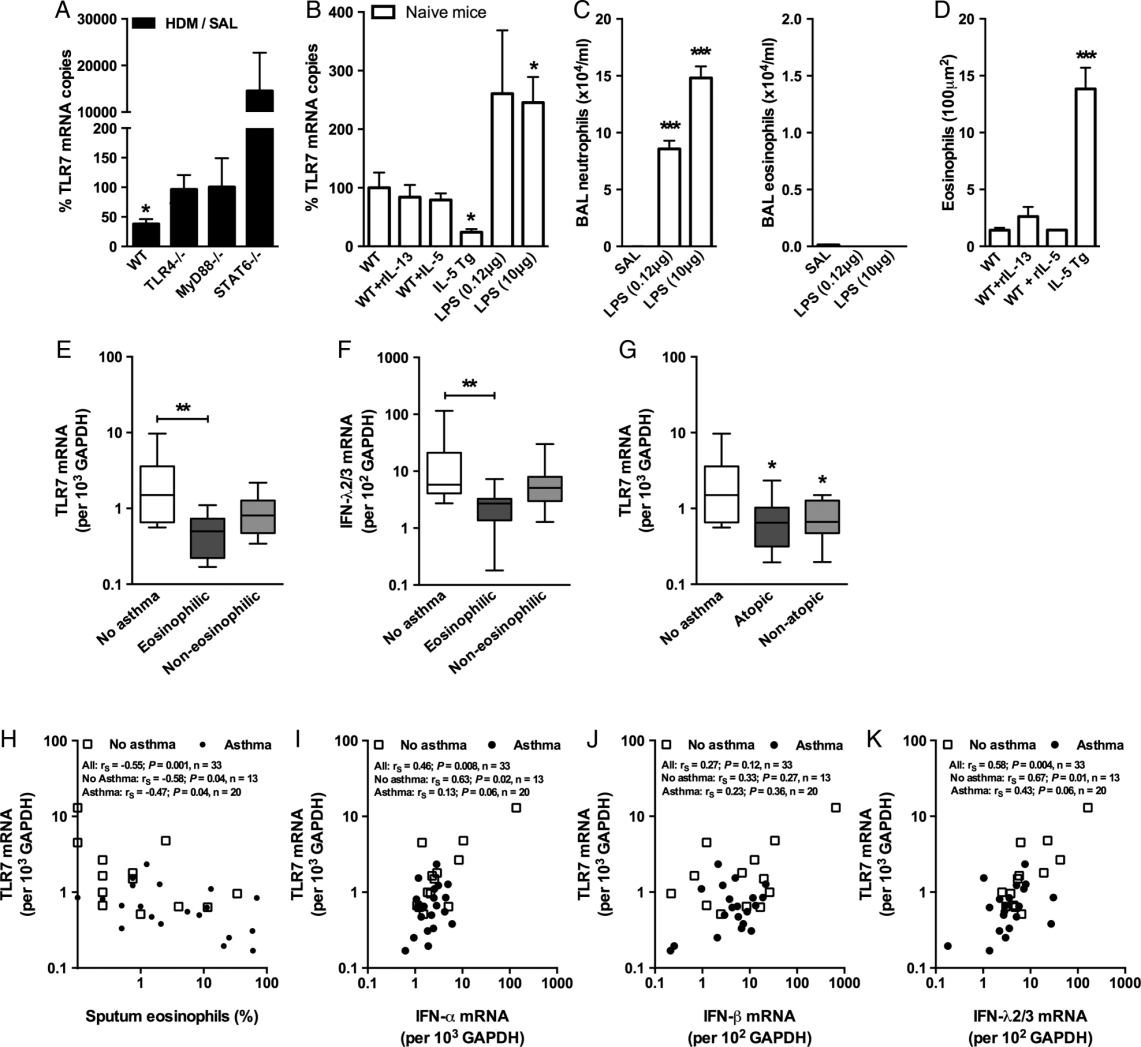
Toll-like receptor (TLR)7 expression is reduced during eosinophilic lung inflammation. (A) Wildtype (WT), *TLR4*^−/−^, *MyD88*^−/−^ and *Stat6*^−/−^ mice were sensitised and challenged with house dust mite (HDM) over 18 days and gene expression of TLR7 in lower airway tissue was quantified by quantitative RT-PCR. TLR7 airway expression in allergic airways as a percentage of sterile saline (SAL) expression for each strain, (B) as well as lung tissue eosinophils and (C) bronchoalveolar lavage (BAL) fluid neutrophils and eosinophils (D) from rIL-5, rIL-13, IL-5 Tg and lipopolysaccharide (LPS)-treated mice 24 h post treatment. Results are mean±SEM (n=4–6 mice per group), gene expression in mice expressed as a % compared to non-allergic SAL-treated wildtype mice. TLR7 (E) and interferon (IFN)λ2/3 (F) expression in bronchial biopsies collected from non-asthmatic (n=13) and asthmatic (n=20) subjects stratified into eosinophilic (>3% eosinophils) or non-eosinophilic (<3% eosinophils) phenotypes based on BAL cell counts. (G) TLR7 expression in bronchial biopsies collected from non-asthmatic (n=13) and asthmatic (n=20) subjects stratified according to atopic status. Lines indicate the median, boxes extend from 25th to the 75th percentile, and error bars extend to 10th and 90th percentiles. (H) Correlation between TLR7 expression from bronchial biopsies and percentage of sputum eosinophils. (I–K) Correlations between TLR7 and IFN expression from patient biopsies. *p<0.05, **p<0.01, ***p<0.001 as determined by Student's t test (A–D) or Kruskal–Wallis with Dunn's multiple comparisons test.

TLR7 expression was assessed in a number of knockout mice strains as it was suppressed in allergic WT mice with T_H_2-mediated AAD (figure 4A). Notably this allergen-induced reduction in TLR7 expression was not observed in TLR4 (*Tlr4*^−/−^), MyD88 (*Myd88*^−/−^) or STAT6 (*Stat6*^−/−^)-deficient mice, suggesting that intact T_H_2-promoting signalling pathways and the presence of eosinophilic airways inflammation are required for suppression of TLR7. To investigate the specific role of T_H_2 cytokines in this in vivo observation, we delivered one dose of recombinant IL-13 or IL-5 intranasally to WT mice and compared those responses to ones challenged with LPS (a TLR4 agonist). Interestingly, in mice that constitutively overexpress IL-5 (*IL-5^Tg/Tg^* mice) TLR7 expression was significantly reduced (figure 4B) and was associated with accumulation of eosinophils in the airways in the absence of allergy (figure 4D). Intranasal administration of one dose of IL-5 or IL-13, however, had no effect on TLR7 expression or eosinophil recruitment although IL-13 did induce AHR and increased expression of Muc5AC (data not shown). In addition, one low or high dose of LPS increased TLR7 expression and resulted in the accumulation of neutrophils but not eosinophils in the lungs (figure 4C,D). Thus eosinophilic airways inflammation due to chronic IL-5 release is associated with a reduction in TLR7 expression while a single administration of IL-5 or IL-13 had no effects on TLR7.

We next analysed TLR7 expression in bronchial biopsies collected from healthy subjects and patients with moderate-to-severe persistent asthma (clinical and demographic data in online supplementary table S2). Importantly, asthmatics with an eosinophilic airways inflammation, as determined by bronchial lavage,^21^ displayed significantly reduced TLR7 (figure 4E) and IFNλ2/3 expression (figure 4F), independent of atopic status (figure 4G). Furthermore, levels of TLR7 expression positively correlated with IFNα and λ2/3 expression (figure 4I–K) and inversely correlated with percentage of sputum eosinophils (figure 4H) but not macrophages or neutrophils (r_s_=0.04; p=0.81; n=33 and r_s_=0.31; p=0.08; n=33, respectively). These results suggest that suppression of TLR7 and IFN expression in moderate-to-severe asthmatics is specifically tied to eosinophilic airways inflammation in this cohort.

## Discussion

Impaired innate IFN responses to respiratory viruses have been proposed as one mechanism underlying the clinical observation of asthmatics being susceptible to RV-induced exacerbation.^7^ ^8^ Innate IFN responses are instigated by a limited number of pattern recognition receptors, such as TLR7 whose cognate ligands include the RV viral genome.^10^ ^11^ Experiments in human immortalised epithelial cell lines (BEAS-2B) have supported a role of TLR3 in mediating an antiviral and anti-inflammatory response.^9^ However, TLR3 expression in the airways^22^ and TLR3-induced responses did not vary in PBMCs derived from asthmatic or atopic patients by comparison to healthy subjects.^22^ We show here, for the first time in vivo, that a lack of TLR7 signalling under conditions that model a viral asthma exacerbation leads to impaired IFN production and exaggerated T_H_2-driven inflammation. Our findings, such as increased levels of the T_H_2-priming cytokines IL-25, IL-33 and TSLP in *Tlr7***^−/−^** mice, mirror those published by Kaiko *et al*,^23^ infecting non-allergic mice with mouse pneumovirus, a mouse pathogen similar to human respiratory syncytial virus. These studies suggest that intact TLR7 signalling is required for sufficient IFN induction and restrainment of proinflammatory responses in the lung.

As TLR7 activation is upstream of IFN production, we hypothesised that administration of exogenous IFN would protect allergic *Tlr7***^−/−^** mice from RV-induced exacerbation. We observed that treatment with type I or III IFNs resulted in the *Tlr7***^−/−^** mice developing a suppressed phenotype similar to that of the WT controls, whose TLR7 signalling is active. All IFN treatments induced the release of IFNγ in the lung, with type I and III IFNs promoting themselves exclusively, indicative of their known distinct signalling pathways.^14^

RV-induced pDC-derived IFN production was dependent upon intact TLR7 signalling in vitro. Additionally, adoptive transfer of TLR7-expressing pDCs into allergic *Tlr7*^−/−^ hosts re-established type I and II IFN responses, limiting RV replication and eosinophilic airways inflammation. Notably TLR7 deficiency did not impair pDC recruitment into the lungs. This implicates activation of TLR7 signalling on pDCs and increasing host IFN release as important therapeutic strategies, either through the use of TLR7 agonists or targeting pathways upstream of IFN production. Other IFN-producing cells may also be relevant. We have recently shown that anti-CCL7 treatment is sufficient to inhibit IRF-7-dependent IFNβ expression in RV infection, which was associated with reduced macrophage inflammation but not pDC influx.^24^ This highlights the complexity of the inflammatory and antiviral immune response on a cellular level, particularly in an allergic environment.

WT allergic mice with T_H_2-driven AAD had significantly lower levels of TLR7 expression compared with non-allergic SAL-treated mice in the absence of RV infection, which is in line with the pattern seen clinically in non-infected eosinophilic asthmatics. TLR4 signalling is required for the development of a robust T_H_2-mediated allergic airways inflammation in response to HDM extract, which also contains low amounts of endotoxins.^19^ Allergic airways inflammation suppresses TLR7 expression, which is prevented by disruption of T_H_2-promoting signalling pathways governed by TLR4, MyD88 and STAT6. In contrast to allergic mice, TLR4 activation by LPS led to an upregulation of TLR7 expression in non-allergic mice. Thus the effects of TLR4 signalling on the regulation of TLR7 expression are determined by the presence or absence of T_H_2-dominant allergic airways inflammation. Interestingly, LPS also upregulated TLR3 expression in human monocytes, which was critical for antiviral responses.^25^ TLR3−/− mice infected with RV1B, however, displayed normal type I IFN responses, unchanged viral titres and reduced inflammatory responses.^10^ This is in marked contrast to our data generated in allergic TLR7−/− mice and previous data in non-allergic TLR7−/− mice.^20^ Further studies are now required to elucidate the clinical effect of endotoxins on TLR7-mediated responses in asthma.

In the absence of T_H_2-dominant allergic airways inflammation, no suppression of TLR7 expression was observed by a single exposure to IL-13 or IL-5. However, chronic exposure to IL-5 alone, which is associated with lung eosinophilia and the development of AHR,^26^ markedly impaired TLR7 lung expression in the absence of allergy. This finding was of clinical relevance because we observed reduced TLR7 expression in endobronchial biopsies from asthmatic patients with eosinophilic inflammatory profiles. Eosinophilic asthmatics also expressed lower levels of innate IFNs in addition to TLR7, a difference that was not observed for expression of TLR3, retinoic acid-inducible gene I or melanoma differentiation-associated gene 5.^27^ These results are congruent with a recent study that mapped TLR7 expression in the airways of severe asthmatics.^28^

**Figure 5:**
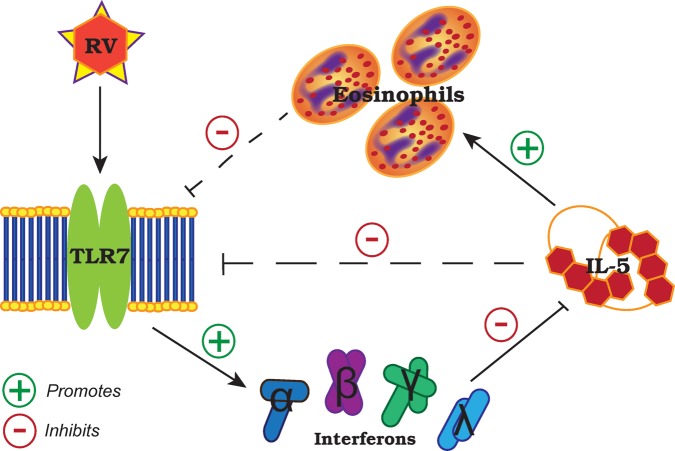
Proposed role and regulation of Toll-like receptor (TLR)7 in rhinovirus infection and allergic airways disease. IL, interleukin; RV, rhinovirus.

Our results suggest a reciprocal regulation between IL-5-induced eosinophilia and TLR7 expression that affects antiviral IFN responses to RV (summary illustrated in figure 5). In the absence of virus, it has been shown that TLR7 stimulation with synthetic agonists in vitro resulted in inhibition of IL-5 through IFNγ and Notch signalling pathways in antigen-presenting cells^29^ and upregulation of IFNγ production by memory CD4+ T cells^30^ and natural killer cells.^31^ Consistent with these findings, we show impaired IFNγ release in the absence of TLR7 promotes T_H_2 immune responses, which can be rescued by type I and III IFN therapy or adoptive transfer of TLR7-sufficient pDCs. This is directly relevant to subjects with moderate-to-severe asthma with a predominantly eosinophilic airways inflammation as these individuals have suppressed—but not fully deficient—TLR7 expression in their lungs, which correlated with reduced IFN expression. Notably, a human monoclonal antibody to IL-5 (mepolizumab) reduced exacerbation frequency in moderate-to-severe asthmatics with a predominantly eosinophilic airways inflammation,^32^ a strategy that may be of specific benefit to that clinical population as not all asthmatics appear to exhibit impaired IFN responses to RV.^33^ Our data suggest that TLR7 and its downstream signalling pathway limits T_H_2 responses in RV-induced asthma exacerbations and may be a promising therapeutic target for the prevention and treatment of viral exacerbation in eosinophilic asthmatics.

## Supplementary Material

Web supplement

Web table 1

Web table 2
