# Supplementary material for: Toll-like receptor 7 governs interferon and inflammatory responses to rhinovirus and is suppressed by IL-5-induced lung eosinophilia
Source: Thorax. 2015 Jun 24;70(9):854–61. doi: 10.1136/thoraxjnl-2014-205465 (PMC4552894; doi:10.1136/thoraxjnl-2014-205465)

Supplementary Figure 1. Expression of T_H2-priming epithelial cytokines IL-25 and TSLP but not IL-33 are higher in exacerbated TLR7-deficient mice. Total RNA was extracted from lower airway tissue of mice following RV-induced exacerbation and expression of IL-25 (A), IL-33 (B), and thymic stromal lymphopoietin (TSLP) (C) assessed by qRT-PCR. Copy numbers are displayed as % compared to WT HDM+UV group. Results are Mean±SEM (n=3-6 mice per group). *, $P < 0.05$, **, $P < 0.01$, ***, $P < 0.001$ as compared to strain-matched HDM+UV group or otherwise indicated as determined by students t-test.

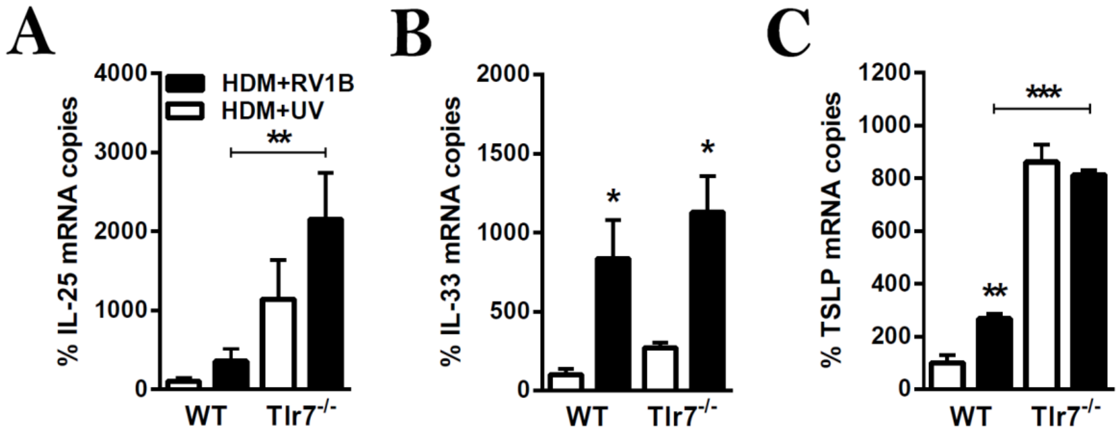

Supplementary Figure 2. TLR7-deficiency leads to heightened local production of chemokines upon RV-induced exacerbation. Lung protein levels of CCL2/MCP1, CCL3/MIP1α and CCL4/MIP1β (A), as well as CXCL9/MIG and CXCL10/IP10 (B) was quantified by bead-based Multiplex Immunoassay in clarified tissue homogenates. (A) CCL7/MCP3 was quantified from the same tissue by sandwich ELISA. Results are Mean±SEM (n=3-4 mice per group) with all protein concentrations normalised to lung weight. *, $P < 0.05$, **, $P < 0.01$, ***, $P < 0.001$ as compared to strain-matched HDM+UV group or otherwise indicated as determined by students t-test.

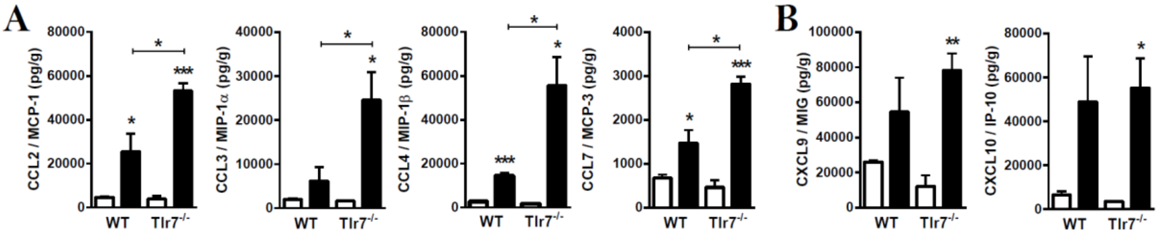

Supplementary Figure 3. TLR7-deficiency does not alter IL-13 production or number of PAS-positive cells following exacerbation. Levels of IL-13 protein and number of PAS$^+$ epithelial cells per 100µm^2 of lung tissue, assessed by ELISA and histological analysis respectively. Data from experiments displayed in Figure 1 (A), Figure 2 (B), and Figure 3 (C). Results are Mean±SEM (n=3-6 mice per group). *, $P < 0.05$, **, $P < 0.01$, ***, $P < 0.001$ as compared to strain-matched HDM+UV group or otherwise indicated as determined by students t-test.

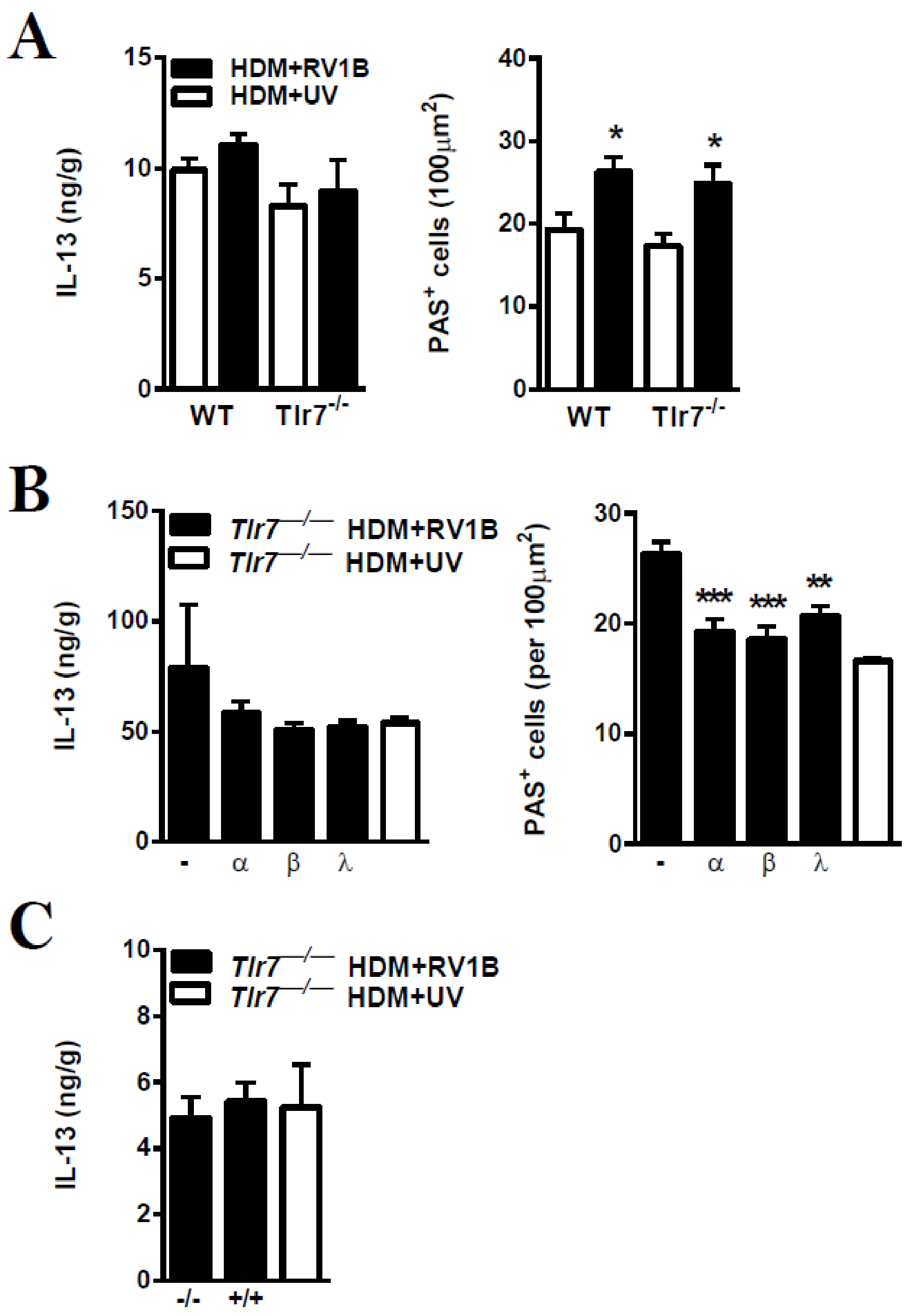

Supplement: Web supplement [file thoraxjnl-2014-205465-s1.pdf]
