# Supplementary material for: Toll-like receptor 7 governs interferon and inflammatory responses to rhinovirus and is suppressed by IL-5-induced lung eosinophilia
Source: Thorax. 2015 Jun 24;70(9):854–61. doi: 10.1136/thoraxjnl-2014-205465 (PMC4552894; doi:10.1136/thoraxjnl-2014-205465)
Supplement: Web table 1 [file thoraxjnl-2014-205465-s2.pdf]

Supplementary Table 1. Primer/probe sequences

Gene	Forward primer (5'-3')	Reverse primer (3'-5')
Murine *HPRT*	AGGCCAGACTTTGTTGGATTTGAA	CAACTTGCGCTCATCTTAGGCTTT
Murine *IL25*	ATGTACCAGGCTGTTGCATTCTTG	CTAAGCCATGACCCGGGGCC
Murine *IL33*	TCCTTGCTTGGCAGTATCCA	TGCTCAATGTGTCAACAGACG
Murine *TSLP*	AGGCTACCCTGAAACTGAG	GGAGATTGCATGAAGGAATACC
Murine *CCL11*	TTCTATTCCTGCTGCTCACGG	AGGGTGCATCTGTTGTTGGTG
Murine *TLR7*	TCCCAACCTTGTTGAGTTGGATTT	TGGGAAGCTTGTGCAATACAGAAA
Human *GAPDH*	ACAGTCAGCCGCATCTTCTTTTG	TAGGCTCTTCATTGGCTCAG
Human *TLR7*	TGGAAATTGCCCTCGTTGTT	TGGTGTAGAAATACTCCTTGATGTG
Human *IFNA*	AGCCCAAGGTTCAGAGTCACCCA	TGTATGTGGGTAGGAGATGGAGAT
Human *IFNB*	AAGAGTTACACTGCCTTTGCCATC	AAAGCAAAGGGCGAGGCCATCGT
Human *IFNL2/3*	CCGGCTCCAGGAGGCCCCAAAAA	CAGGTTGCATGACTGGTGGGAGGG
(+)-strand hRV1B	AGTCCTCCGGCCCCTGAATG	AAAGTAGTTGGTCCATCCCGC

HPRT = Hypoxanthine-quanine phosphoribosyltransferase

GAPDH = Glyceraldehyde-3-phosphate dehydrogenase
