# Supplementary material for: Toll-like receptor 7 governs interferon and inflammatory responses to rhinovirus and is suppressed by IL-5-induced lung eosinophilia
Source: Thorax. 2015 Jun 24;70(9):854–61. doi: 10.1136/thoraxjnl-2014-205465 (PMC4552894; doi:10.1136/thoraxjnl-2014-205465)
Supplement: Web table 2 [file thoraxjnl-2014-205465-s3.pdf]

Supplementary Table 2 online**: Characteristics of healthy and asthmatic subjects.**

	Healthy	Asthmatic
N	13	20
Age (years)	50.8 (26, 69)	57.8 (30, 77)
Males, n (%)	6 (46.2)	4 (25)
Non-smoker, n (%)	13 (100)	17 (85)
FEV_1% predicted	98.4 ± 4.1	67.4 ± 5.4***
ICS, n (%)	0 (0)	20 (100)
Mean ICS dose – (µg, Beclometasone equivalent)	0	505.26
LABA, n (%)	0 (0)	20 (100)
OCS, n (%)	0 (0)	1 (7.1)

***, $P < 0.001$
